# scRepertoire: An R-based toolkit for single-cell immune receptor analysis

**DOI:** 10.12688/f1000research.22139.2

**Published:** 2020-06-15

**Authors:** Nicholas Borcherding, Nicholas L. Bormann, Gloria Kraus

**Affiliations:** 1 Department of Pathology, University of Iowa, Iowa City, IA, USA; 2 Medical Scientist Training Program, University of Iowa, Iowa City, IA, USA; 3 Cancer Biology Graduate Program, University of Iowa, Iowa City, IA, USA; 4 Holden Comprehensive Cancer Center, University of Iowa, Iowa City, IA, USA; 5 Department of Psychiatry, University of Iowa, Iowa ity, IA, USA; 6 Faculty of Medicine, Center for Regenerative Therapies Dresden, Technische Universität Dresden, Dresden, Germany

**Keywords:** Single-cell RNA sequencing, immune receptor profiling, R, clonotypic analysis

## Abstract

Single-cell sequencing is an emerging technology in the field of immunology and oncology that allows researchers to couple RNA quantification and other modalities, like immune cell receptor profiling at the level of an individual cell. A number of workflows and software packages have been created to process and analyze single-cell transcriptomic data. These packages allow users to take the vast dimensionality of the data generated in single-cell-based experiments and distill the data into novel insights. Unlike the transcriptomic field, there is a lack of options for software that allow for single-cell immune receptor profiling. Enabling users to easily combine mRNA and immune profiling, scRepertoire was built to process data derived from 10x Genomics Chromium Immune Profiling for both T-cell receptor (TCR) and immunoglobulin (Ig) enrichment workflows and subsequently interacts with a number of popular R packages for single-cell expression, such as Seurat. The scRepertoire R package and processed data are open source and available on GitHub and provides in-depth tutorials on the capability of the package.

## Introduction

The molecular resolution offered by single-cell sequencing (SCS) technologies has led to extensive investigations in the realms of developmental biology, oncology, and immunology. In terms of the latter field, SCS offers the ability to couple the exploration of transcriptomic heterogeneity in immune cells along a disease process with clonality ^1^. A number of methods exist for dimensional reduction of mRNA data, reviewed by Chen *et al.* ^2^ that have been implemented into R packages to assist in processing and analysis of SCS experiments. However, a gap exists in the processing of V(D)J sequencing, descriptive statistics, clonal comparisons, and repertoire diversity with the current SCS R packages.

**Figure 1.**
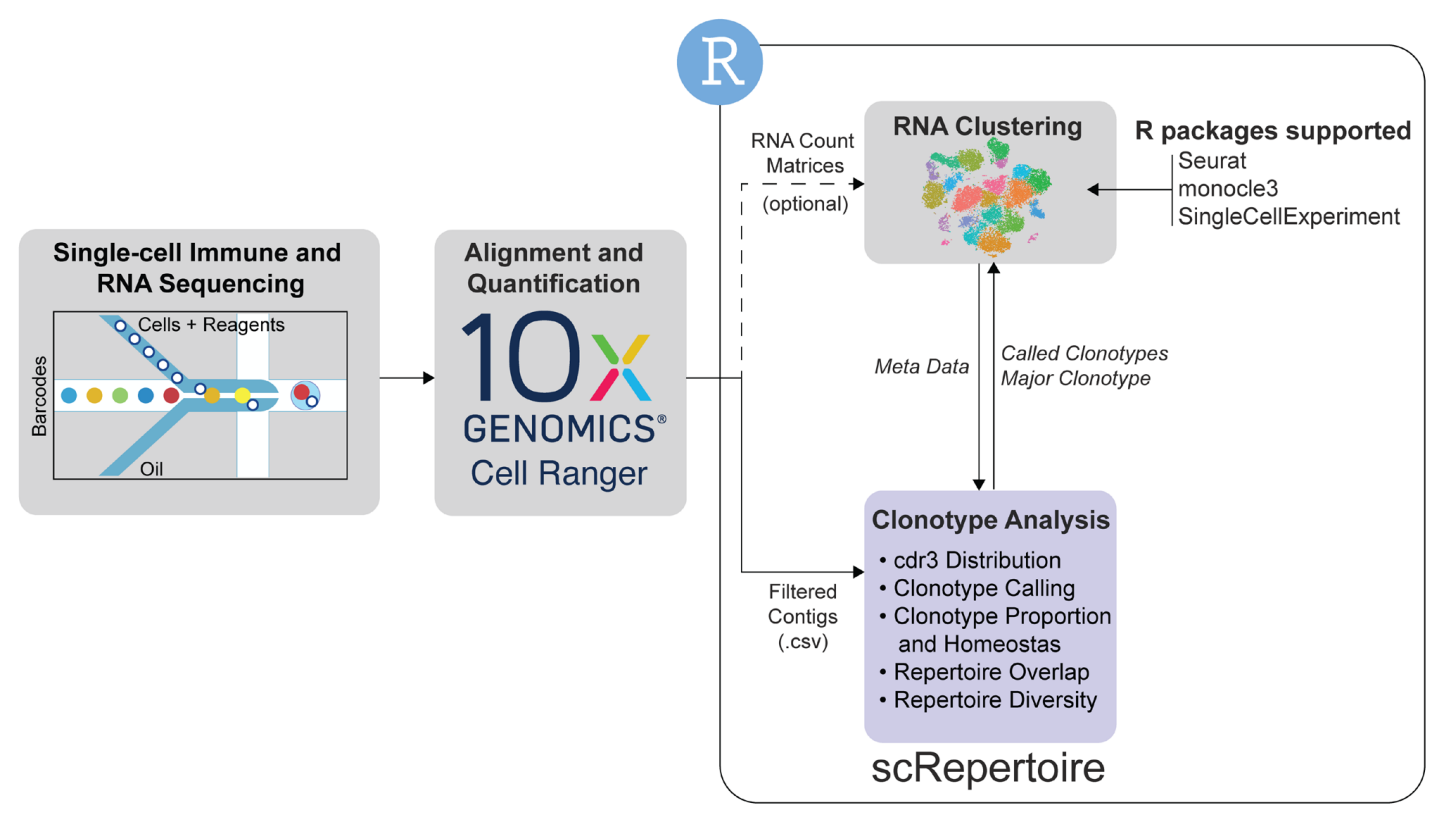
A general workflow for single-cell data analysis involving scRepertoire. The analysis starts with the single-cell immune and mRNA sequencing and Cell Ranger-based alignment with the 10x Genomics pipeline. With the TCR or Ig sequencing, scRepertoire can import the filtered overlapping DNA segments, or contigs. The alignments are filtered by cell type of interest and combined using the individual cell barcodes. Clonotypes can be called using the gene sequence of the immune receptor loci, CDR3 nucleotide sequence or CDR3 amino acid sequence. After clonotype assignment, more extensive clonotypic analysis can be performed at the individual sample level or across all samples. General outputs from scRepertoire can be imported into a number of single-cell expression formats to visualize clonotype data overlaid onto the cell clustering. Likewise, metadata from the expression objects can be imported into scRepertoire to analyze clonotypes by assigned clusters.

With these limitations in mind, scRepertoire ^3^ was generated ( Figure 1). Built using R, scRepertoire is a toolkit to assist in the analysis of immune profiles for both B and T cells, while interacting with the popular Seurat pipeline ^4– 6^, as well as SingleCellExperiment and monocle3 class expression objects. scRepertoire also includes processed single-cell mRNA and V(D)J sequencing data of 12,911 tumor-infiltrating and peripheral-blood T cells derived from three renal clear cell carcinoma patient, which is characterized below to demonstrate the capabilities of the package.

## Methods

### Operation

System requirements for running scRepertoire ^3^ include the installation of R v3.5.1 and the the Seurat R package (v3.1.2). Utilization of scRepertoire is dependent on the total number of single-cells being processed, with a base estimate of 1 Gb of random-access memory and a modern CPU.

### Data

The isolation and processing of the 10x-Genomics-based single-cell mRNA and V(D)J Chromium sequencing data for immune cells has previously been described ^7, 8^. In addition, T cells were identified using expression values for canonical T cell markers: *CD3D*, *CD4*, *CD8A*, *CD8B1* and previous clustering. T cells were isolated and reclustered using the integration method from the Seurat R package (v3.1.2) with 20 principal components and a resolution of 0.5 ^4^. All code used to generate the figures appearing in the manuscript is available at https://github.com/ncborcherding/scRepertoire.

### Implementation

The scRepertoire was built and tested in R v3.5.1. Analysis for scRepertoire was inspired from the bulk immune profiling tcR (v2.2.4) R package without derivations in code ^9^. Clonotypes can be called using the combination of immune loci genes, a more sensitive approach, or the nucleotide/amino acid sequence of the complementary-determining region 3 (CDR3). In addition to the base functions in R, data processing was performed using the dplyr (v0.8.3) and reshape2 (v1.4.3) R packages. Visualizations are generated using the ggplot2 (v3.2.1) and ggalluvial (v0.11.1) R packages with color pallets derived from the use of colorRamps (v2.3) and RColorBrewer (v1.1.2) R packages. Diversity metrics are calculated using the vegan (v2.5-6) R package. Visual outputs of functions are stored as layers of geometric or statistical ggplot layering, allowing users to easily modify presentation.

## Results

### Clonal analysis

**Figure 2.**
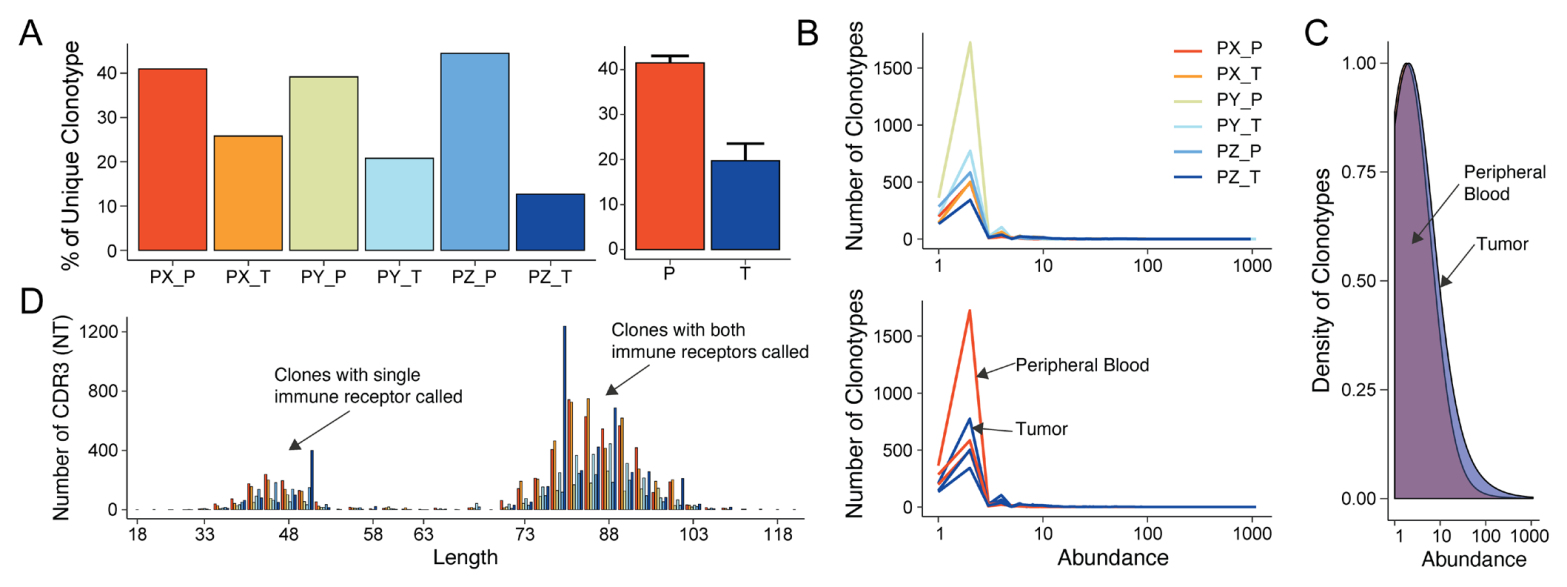
Basic clonotypic analysis functions in scRepertoire. ( **A**) Scaled unique clonotypes by total number of TCRs sequenced by patient and type of sample (peripheral, P; tumor, T), using the *quantContig* function. ( **B**) Total abundance of clonotypes by sample and type using the *abundanceContig* function. ( **C**) Relative abundance of clonotypes using density comparing peripheral blood to tumor samples. ( **D**) CDR3 nucleotide length analysis by sample using the *lengthContig* function. The bimodal nature of the curve is a function of calling clonotypes for cells with both one and two immune receptors sequenced.

scRepertoire ^3^ can be used to call clonotypes using the CDR3 amino acid/nucleotide sequences, by gene usage, or by the combination of CDR3 nucleotide sequences and genes. Using the *quantContig* function, unique clonotypes can be visualized as raw values or scaled to the size of the library for samples or by type ( Figure 2A). The total abundance of clonotypes can also be visualized calling *abundanceContig* ( Figure 2B) or relative abundance of clonotypes ( Figure 2C). Additionally, the distribution of CDR3 nucleotide or amino acid sequences for clonotypes can be visualized with *lengthContig* ( Figure 2D). More advance distribution analysis is also available using the *clonesizeDistribution* function based on recent work using Jensen-Shannon divergence.

### Proportional analysis and diversity measures

**Figure 3.**
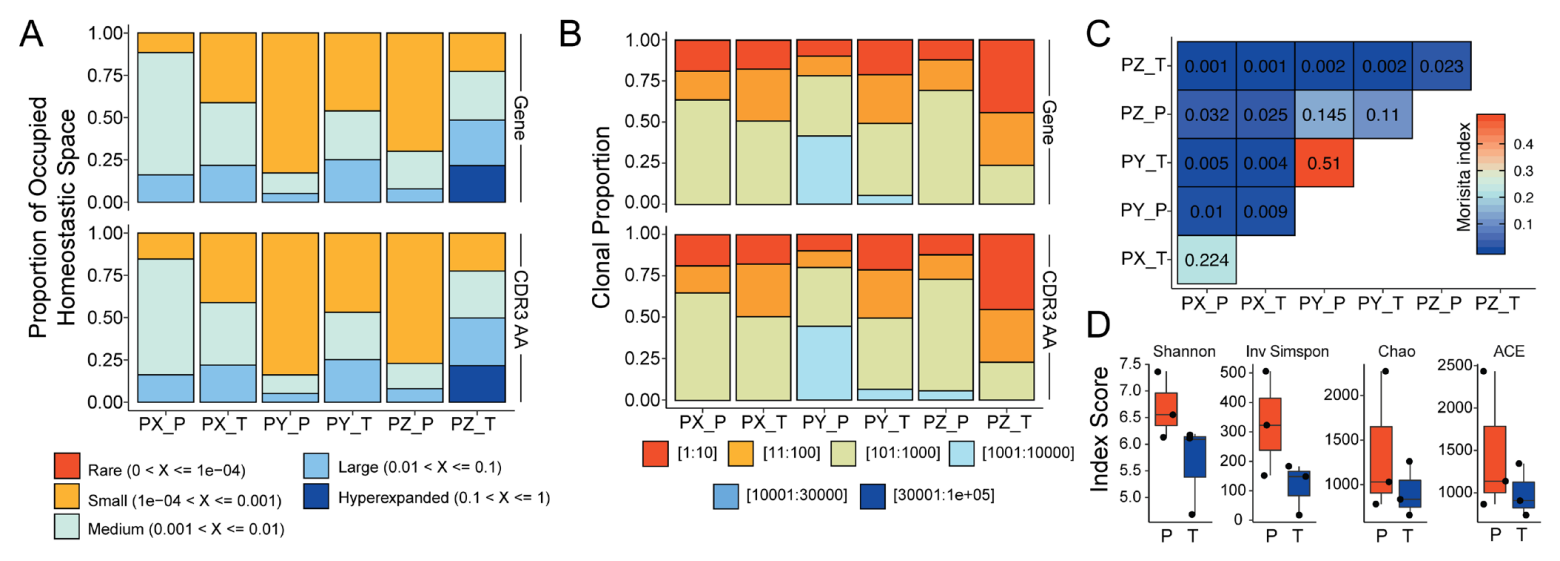
Advanced clonal measures between samples. ( **A**) Clonal homeostatic space representations across all six samples using the gene and CDR3 AA sequence for clonotype calling. ( **B**) Relative proportional space occupied by specific clonotypes across all six samples using the gene and CDR3 AA sequence for clonotype calling. ( **C**) Morisita overlap quantifications for clonotypes across all six samples. ( **D**) Diversity measures based on clonotypes by sample type using Shannon, Inverse Simpson, Chao, and abundance-based coverage estimator (ACE) indices.

More in depth analysis of clonal architecture is available. Within the framework of scRepertoire, analysis of clonal homeostasis, or the clonal space occupied by clonotypes of specific proportions, can be visualized by *clonalHomeostasis* function ( Figure 3A). Similarly, *clonalProportion* can be called to look at the proportion of clonal space occupied by specific clonotypes ( Figure 3B). Overlap between the samples can be calculated and visualized with *clonalOverlap*, using either the overlap coefficient or Morisita index methods ( Figure 3C). Measured of diversity across samples or groups can be quantified with the *clonalDiversity* function, demonstrating an overall reduction in clonal diversity in tumor samples ( Figure 3D).

### Expression interaction

**Figure 4.**
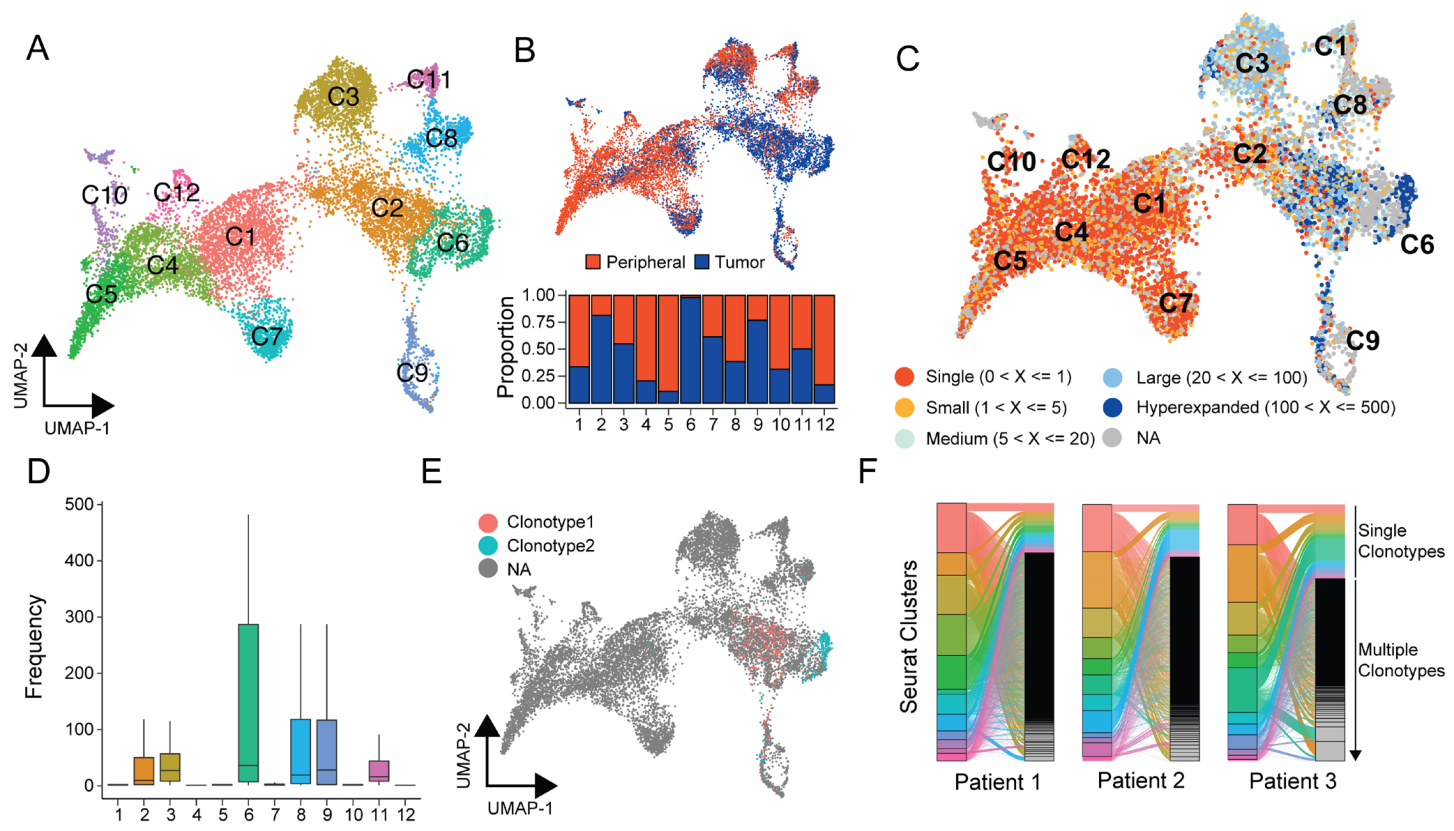
Interaction of scRepertoire with the single-cell expression R packages. ( **A**) UMAP projection from Seurat of the ccRCC T cells (n=12,911) into 12 distinct clusters. ( **B**) UMAP projection with peripheral blood (red) and tumor (blued) populations highlighted and an accompanying relative proportion composition of each cluster, scaled by the total number of peripheral blood and tumor cells, respectively. ( **C**) Using the *combineExpression* function places individual cells into groups by the number of clonotypes, which then can be displayed overlaid with the UMAP projection. ( **D**) After combining the clonotype information with the Seurat object, *highlightClonotypes* can be used to specifically highlight the individual clonotypes of interest using the sequence information. ( **E**) Interaction of clonotypes between multiple categories can be examined using the *alluvialClonotypes* function.

After the processing and analysis of the TCR repertoire with the base features, the next step is using scRepertoire to interact with the single-cell mRNA data. The expression data for the 12,911 cells built into the package have already been clusters ( Figure 4A), with a clear distribution of the clusters into peripheral-blood- versus tumor-predominant ( Figure 4B). Using the *combineExpression* function in scRepertoire, we can look at the clonotypic frequencies of cells that comprise the UMAP-based clusters ( Figure 4C). This function also works with the SingleCellExperiment and monocle3 class of expression objects. ( Figure 4D). In addition to clonal distribution, we can also use *highlightClonotypes* to set specific sequences of clonotypes to be visualized ( Figure 4D), with clonotype 1 referring to the amino acid sequence “CAVNGGSQGNLIF_CSAEREDTDTQYF” and clonotype 2 for the amino acid sequence "NA_CATSATLRVVAEKLFF". Interesting clonotype 2 is restricted to a subcluster of the C6 cluster ( Figure 4D). After combining both the clonotype and expression data, interaction between categories, such as cluster label and clonotype frequency can be visualized with the *alluvialClonotypes* function ( Figure 4E). This function can also be used to examine the dynamics of single or multiple expanded clonotypes across the categorical variables ( Figure 4E). Further, after the attachment of the expression information to a single-cell expression object, the function, *expression2List()* allows users generate analyses based on any categorical variable in the meta data..

## Conclusions

scRepertoire ^3^ is a R-based toolkit for the analysis of single-cell immune receptor profiling. The package is able to take the annotated filtered outputs from the 10x Genomics Cell Ranger platform and provide analysis a number of modalities, including calling clonotypes, clonal space/homeostasis, clonal diversity, and repertoire overlap between samples. Outputs from scRepertoire can combined with dimensional reduction strategies for single-cell RNA quantifications, allowing users to analyze mRNA and immune profiles together. Visualization functions in scRepertoire have a parameter, exportTable, allowing users to examine the quantifications underlying the generation of the graphs. Under the creative commons v4.0 license, the scRepertoire package is freely available from the GitHub repository and is extensively annotated to assist in implementation and modification.

## Data availability

### Source data

Zenodo: scRepertoire. https://doi.org/10.5281/zenodo.3856827 ^3^.

Folder ‘Data’ contains all data required to run the vignettes described in the *Results*. This is also available on GitHub.

Data are available under the terms of the Creative Commons Attribution 4.0 International license (CC-BY 4.0).

## Software availability

Source code is available from GitHub: https://github.com/ncborcherding/scRepertoire.

Archived source code at the time of publication: https://doi.org/10.5281/zenodo.3856827 ^3^.

License: Creative Commons Attribution 4.0 International.

## References

[1] Papalexi E Satija R: Single-cell RNA sequencing to explore immune cell heterogeneity. *Nat Rev Immunol.* 2018;18(1):35–45. 10.1038/nri.2017.76 28787399

[2] Chen G Ning B Shi T: Single-Cell RNA-Seq Technologies and Related Computational Data Analysis. *Front Genet.* 2019;10:317. 10.3389/fgene.2019.00317 31024627 PMC6460256

[3] Borcherding N Bormann NL: scRepertoire (Version 1.2.0). *Zenodo.* 2020 10.5281/zenodo.3856827 PMC7400693 32789006

[4] Stuart T Butler A Hoffman P: Comprehensive Integration of Single-Cell Data. *Cell.* 2019;177(7):1888–1902.e21. 10.1016/j.cell.2019.05.031 31178118 PMC6687398

[5] Macosko EZ Basu A Satija R: Highly Parallel Genome-wide Expression Profiling of Individual Cells Using Nanoliter Droplets. *Cell.* 2015;161(5):1202–14. 10.1016/j.cell.2015.05.002 26000488 PMC4481139

[6] Butler A Hoffman P Smibert P: Integrating single-cell transcriptomic data across different conditions, technologies, and species. *Nat Biotechnol.* 2018;36(5):411–420. 10.1038/nbt.4096 29608179 PMC6700744

[7] Borcherding N Ahmed KK Voigt AP: Transcriptional heterogeneity in cancer-associated regulatory T cells is predictive of survival. *bioRxiv.* 2018; 478628. 10.1101/478628

[8] Vishwakarma A Bocherding N Chimenti MS: Mapping the Immune Landscape of Clear Cell Renal Cell Carcinoma by Single-Cell RNA-seq. *bioRxiv.* 2019; 824482. 10.1101/824482

[9] Nazarov VI Pogorelyy MV Komech EA: tcR: an R package for T cell receptor repertoire advanced data analysis. *BMC Bioinformatics.* 2015;16:175. 10.1186/s12859-015-0613-1 26017500 PMC4445501

