# Supplementary material for: scRepertoire: An R-based toolkit for single-cell immune receptor analysis
Source: F1000Res. 2020 Jun 15;9:47. Originally published 2020 Jan 27. [Version 2] doi: 10.12688/f1000research.22139.2 (PMC7400693; doi:10.12688/f1000research.22139.2)
Supplement: Peer review history

# Peer review: Reviewer response for version 2

**DOI:** 10.5256/f1000research.27282.r64820

**Published:** 2020-08-03

**Authors:** Massimiliano Pagani, Raoul Jean Pierre Bonnal, Lorenzo Drufuca

**Affiliations:** 1 National Institute of Molecular Genetics, Milan, Italy; 2 School of Medicine and Surgery, Università degli Studi di Milano Bicocca, Milan, Italy

I can confirm that the authors addressed all the issues and the paper is now suitable for indexing.

Are the conclusions about the tool and its performance adequately supported by the findings presented in the article?

Yes

Is the rationale for developing the new software tool clearly explained?

Partly

Is the description of the software tool technically sound?

Partly

Are sufficient details of the code, methods and analysis (if applicable) provided to allow replication of the software development and its use by others?

Yes

Is sufficient information provided to allow interpretation of the expected output datasets and any results generated using the tool?

Yes

Reviewer Expertise:

NA

We confirm that we have read this submission and believe that we have an appropriate level of expertise to confirm that it is of an acceptable scientific standard.

# Peer review: Reviewer response for version 2

**DOI:** 10.5256/f1000research.27282.r64821

**Published:** 2020-07-09

**Authors:** Tim Stuart

**Affiliations:** 1 New York Genome Center, New York City, NY, USA

The authors have now addressed all my original comments.

Are the conclusions about the tool and its performance adequately supported by the findings presented in the article?

Yes

Is the rationale for developing the new software tool clearly explained?

Partly

Is the description of the software tool technically sound?

Yes

Are sufficient details of the code, methods and analysis (if applicable) provided to allow replication of the software development and its use by others?

Yes

Is sufficient information provided to allow interpretation of the expected output datasets and any results generated using the tool?

Yes

Reviewer Expertise:

Single-cell genomics, bioinformatics, epigenomics

I confirm that I have read this submission and believe that I have an appropriate level of expertise to confirm that it is of an acceptable scientific standard.

# Peer review: Reviewer response for version 1

**DOI:** 10.5256/f1000research.24415.r60873

**Published:** 2020-03-09

**Authors:** Tim Stuart

**Affiliations:** 1 New York Genome Center, New York City, NY, USA

This paper presents a new R package for the analysis of T-cell and B-cell clonotypes inferred from V(D)J recombination events. The package is designed for the analysis of data generated using the 10x Genomics V(D)J kit that allows clonotypes and transcriptomes to be co-assayed in the same individual cells.

 Overall the package appears to fill an unmet need in the analysis of single-cell V(D)J data, and works nicely with the existing Seurat package for single-cell analysis.

 While the focus of the paper is on the software, a more detailed explanation of the biology of V(D)J recombination and how this information is detected in single cells would improve the clarity of the manuscript. A description of the major existing challenges and goals of immune clonotype analysis could also be added.

 I do not have extensive expertise in the immunology and so my review focuses on the software implementation. I leave discussion of the value and impact of the software to other reviewers. However I will reiterate comments from other reviewers in saying that there should be a distinction made between T and B cell clonotypes, due to the Ig class switching that occurs in B cells. 

 The paper also states that “The expression data for the 12,911 cells built into the package”. I believe the clonotype information is included in the package in the data directory, but I don’t think the expression data is included. Including large gene expression matrix within the R package is not advisable anyway, and the authors should make it available through other means (NCBI GEO, for example).

**Code review**

 I would encourage the authors to submit the package to CRAN or another R repository. This assist in the distribution of the package, and the process of getting the package accepted on CRAN will likely greatly improve the code quality. At a minimum, the authors should try to get the package to pass R CMD check with no notes, warnings, or errors.

 Currently the package includes all rendered figures in the vignettes folder. I would recommend removing these files, and also removing the `ggsave` function calls within the vignette to prevent these files being written while compiling the vignette. This will reduce the size of the package.

 Several functions in the package use scoping assignment to assign variables in the global environment. This is considered bad practice and should be avoided in all cases.

 Extra files such as .DS_Store should be removed from the git repository and from the package. Use the .gitignore and .Rbuildignore files for this.

 Avoid importing code within R functions, for example `require(ggplot2)` calls. Instead, document the dependencies using roxygen2, for example `@importFrom ggplot2 ggplot`. 

 To access data in a Seurat object, I highly recommend using the functions defined in Seurat for this purpose rather than accessing the slots directly. For example, use obj[[]] to access metadata rather than obj@meta.data and Idents(obj) rather than obj@active.ident

 In general the documentation of functions can be greatly improved. Try to include a text description of each function, a detailed description of the parameters, document the returned values, and include an executable example.

 It is generally not advisable to overwrite functions in base R or other packages with variable names, for example the `call` variable in `clonalDiversity` overwrites the base R `call` function.

 Replace code like `class(df)[1] == “Seurat”` with `inherits(x = df, what = "Seurat”)`

 In plotting functions such as clonalOverlap, consider returning the ggplot object rather than printing the object. For example, replace `suppressWarnings(print(plot))` with `return(plot)`. This will allow users to modify the plot that is generated. 

 Some code sections are duplicated, for example L91:104 and L131:144 in seuratFunctions.R. Consider putting duplicated code into functions.

 Imported functions should be added to the namespace. Documenting the imports using roxygen2 (as has been done for parameters and exports) will take care of this.

 The highlightClonotypes function is a bit redundant with existing functions in Seurat, ie DimPlot function with the cells.highlight parameter.

Are the conclusions about the tool and its performance adequately supported by the findings presented in the article?

Yes

Is the rationale for developing the new software tool clearly explained?

Partly

Is the description of the software tool technically sound?

Yes

Are sufficient details of the code, methods and analysis (if applicable) provided to allow replication of the software development and its use by others?

Yes

Is sufficient information provided to allow interpretation of the expected output datasets and any results generated using the tool?

Yes

Reviewer Expertise:

Single-cell genomics, bioinformatics, epigenomics

I confirm that I have read this submission and believe that I have an appropriate level of expertise to confirm that it is of an acceptable scientific standard, however I have significant reservations, as outlined above.

# Response

**Published:** 2020-06-09

**Authors:** Nicholas Borcherding

**Affiliations:** University of Iowa, USA

This paper presents a new R package for the analysis of T-cell and B-cell clonotypes inferred from V(D)J recombination events. The package is designed for the analysis of data generated using the 10x Genomics V(D)J kit that allows clonotypes and transcriptomes to be co-assayed in the same individual cells.

 Overall the package appears to fill an unmet need in the analysis of single-cell V(D)J data, and works nicely with the existing Seurat package for single-cell analysis.

**While the focus of the paper is on the software, a more detailed explanation of the biology of V(D)J recombination and how this information is detected in single cells would improve the clarity of the manuscript. A description of the major existing challenges and goals of immune clonotype analysis could also be added.**

 The authors agree that a more extensive explanation of VDJ biology would be a benefit, however, due to the limitations of less than 1000 words and 10 citations, we are limited by the structure of the F1000Research. We have added citations to the readme file to help users.

 I do not have extensive expertise in the immunology and so my review focuses on the software implementation. I leave discussion of the value and impact of the software to other reviewers. However I will reiterate comments from other reviewers in saying that there should be a distinction made between T and B cell clonotypes, due to the Ig class switching that occurs in B cells. 

**The paper also states that “The expression data for the 12,911 cells built into the package”. I believe the clonotype information is included in the package in the data directory, but I don’t think the expression data is included. Including large gene expression matrix within the R package is not advisable anyway, and the authors should make it available through other means (NCBI GEO, for example).**

 The seurat object with the package is available in the zenodo repository and linked in the github repository. The raw data has been deposited in GEO and more information can be found in the two preprints cited.

 Code review

**I would encourage the authors to submit the package to CRAN or another R repository. This assist in the distribution of the package, and the process of getting the package accepted on CRAN will likely greatly improve the code quality. At a minimum, the authors should try to get the package to pass R CMD check with no notes, warnings, or errors.**

 Thank you for the suggestions, the authors believe this point helped the structure of the package immensely. We have used the devtools check function and as of the version in the github repository and zenodo at resubmission, there is no warnings, errors, or notes. We have begun the process of submitting the package to Bioconductor and have passed the build phase of the process.

**Currently the package includes all rendered figures in the vignettes folder. I would recommend removing these files, and also removing the `ggsave` function calls within the vignette to prevent these files being written while compiling the vignette. This will reduce the size of the package.**

 Thank you for the suggestion, this has been completed.

**Several functions in the package use scoping assignment to assign variables in the global environment. This is considered bad practice and should be avoided in all cases.**

 Scoping assignments in these instances are to give users the options of exporting the tables or matrices that form the visualization. We have modified the activity of the exportTable parameter to return a data frame used if TRUE or visualization if FALSE (default).

**Extra files such as .DS_Store should be removed from the git repository and from the package. Use the .gitignore and .Rbuildignore files for this.**

 Thank you for the suggestion, we have removed .DS_Store.

**Avoid importing code within R functions, for example `require(ggplot2)` calls. Instead, document the dependencies using roxygen2, for example `@importFrom ggplot2 ggplot`.** 

 Thank you for the suggestion, we have made this change.

**To access data in a Seurat object, I highly recommend using the functions defined in Seurat for this purpose rather than accessing the slots directly. For example, use obj[[]] to access metadata rather than obj@meta.data and Idents(obj) rather than** **obj@active.ident**



Thanks for the suggestion, we have changed this in the new version of the R package.



**In general the documentation of functions can be greatly improved. Try to include a text description of each function, a detailed description of the parameters, document the returned values, and include an executable example.**



Both reviewers have made this excellent suggestion, the new version of scRepertoire has more comprehensive documentation, including descriptions and working examples.



**It is generally not advisable to overwrite functions in base R or other packages with variable names, for example the `call` variable in `clonalDiversity` overwrites the base R `call` function.**



Thank you for the suggestion, we have modified this for all functions.



**Replace code like `class(df)[1] == “Seurat”` with `inherits(x = df, what = "Seurat”)`**

We have made this change and also replace all class() evaluations.



**In plotting functions such as clonalOverlap, consider returning the ggplot object rather than printing the object. For example, replace `suppressWarnings(print(plot))` with `return(plot)`. This will allow users to modify the plot that is generated.**



Excellent suggestion and this has been fixed.



**Some code sections are duplicated, for example L91:104 and L131:144 in seuratFunctions.R. Consider putting duplicated code into functions.**



Thank you for the suggestion, part of the change in the “call” to cloneCall variable in the functions also including creating a function to deal with the user selection. These lines were consistently repeated across multiple functions, but now have been reduced. We have also extensively modularized the code and added these to utils.R .



**Imported functions should be added to the namespace. Documenting the imports using roxygen2 (as has been done for parameters and exports) will take care of this.**



This has been completed.



**The highlightClonotypes function is a bit redundant with existing functions in Seurat, ie DimPlot function with the cells.highlight parameter.**



Agreed, although redundant, this was a request from an early beta tester, we plan to keep the function in.

# Peer review: Reviewer response for version 1

**DOI:** 10.5256/f1000research.24415.r59209

**Published:** 2020-03-03

**Authors:** Massimiliano Pagani, Lorenzo Drufuca, Raoul Jean Pierre Bonnal

**Affiliations:** 1 School of Medicine and Surgery, Università degli Studi di Milano Bicocca, Milan, Italy; 2 National Institute of Molecular Genetics, Milan, Italy

The paper describes a new package (scRepertoire) of functions for the analysis of clonality in single cell experiments in the R analytical environment.

 While it is true, as it is claimed, that 'a gap exists in the processing of V(D)J sequencing, descriptive statistics, clonal comparisons, and repertoire diversity with the current SCS R packages', nonetheless few packages already exist for the analysis of clonality in bulk samples (such as the inspiring package tcR). From the text it is not immediately clear why there is the need for a brand new package rather than a simpler 'wrapper' to bridge the gap between single cell based data and existing analytical packages.

 Although package functionalities are clearly illustrated in the paper, software documentation could be improved: behaviour of some functions is not immediately clear without studying the code directly (e.g. the way multi-chains clonotypes are treated by combineContigs()).

 Similar to its inspiring package (tcR) and leveraging on the vegan package, scRepertoire implements different measures for diversity. Nonetheless, it has been suggested that conventional diversity measures fail to describe properly clonotype distributions and several complementary methods have been proposed like the Recon package by Kaplinsky ^1^ and Arnaout ^1^ or Startrac package by Zhang and colleagues ^2^. Interaction with such packages would greatly increase scRepertoire analytical effectiveness.

 Similarly, the function abundanceContig(), especially in the unscaled form, is of little use per se. Interestingly, recent methods have been proposed to for the comparative analysis of clone size distributions ^3^ and could be easily incorporated into scRepertoire adding considerable power to it (though assessment of required numerosity should be introduced).

 The package claims to be designed both for TCR and BCR analysis but definition of clonality in B cells is slightly different than in T cells due to isotype switch and somatic hypermutation phenomena following activation. Therefore clonotype identity between two cells should be defined differently between BCR and TCR analysis.

 Concerning overlap, beside the nice representation as a heatmap, it could be useful to have the chance to output the matrix itself rather than the plot only.

 10x vdj methods occasionally fails to reconstruct complete clonotypes or it reconstructs putatively aberrant clonotypes (clonotypes with multiple beta chains). Currently scRepertoire does not allow to filter for specific chain compositions but such feature would be worth adding, together with a graphical visualization of relative frequencies of chain composition across clonotypes.

 Paired 10x gene expression profile and vdj scoped analysis are not guaranteed to reconstruct the information for the exact same pool of barcodes, thus the combineSeurat() function could be improved by allowing to specify whether an inner or Seurat-sided joining is to be performed and ensuring that the joining is performed correctly.

Are the conclusions about the tool and its performance adequately supported by the findings presented in the article?

Yes

Is the rationale for developing the new software tool clearly explained?

Partly

Is the description of the software tool technically sound?

Partly

Are sufficient details of the code, methods and analysis (if applicable) provided to allow replication of the software development and its use by others?

Yes

Is sufficient information provided to allow interpretation of the expected output datasets and any results generated using the tool?

Yes

Reviewer Expertise:

Integrative Biology, Cancer Immunology

We confirm that we have read this submission and believe that we have an appropriate level of expertise to confirm that it is of an acceptable scientific standard, however we have significant reservations, as outlined above.

## References

[1] : Robust estimates of overall immune-repertoire diversity from high-throughput measurements on samples. *Nature Communications*.2016;7(1) : 10.1038/ncomms11881 10.1038/ncomms11881 PMC491262527302887

[2] : Lineage tracking reveals dynamic relationships of T cells in colorectal cancer. *Nature*.564(7735) : 10.1038/s41586-018-0694-x 268-272 10.1038/s41586-018-0694-x 30479382

[3] : powerTCR: A model-based approach to comparative analysis of the clone size distribution of the T cell receptor repertoire. *PLoS Comput Biol*.14(11) : 10.1371/journal.pcbi.1006571 e1006571 10.1371/journal.pcbi.1006571 30485278PMC6287877

# Response

**Published:** 2020-06-09

**Authors:** Nicholas Borcherding

**Affiliations:** University of Iowa, USA

The paper describes a new package (scRepertoire) of functions for the analysis of clonality in single cell experiments in the R analytical environment.

**While it is true, as it is claimed, that 'a gap exists in the processing of V(D)J sequencing, descriptive statistics, clonal comparisons, and repertoire diversity with the current SCS R packages', nonetheless few packages already exist for the analysis of clonality in bulk samples (such as the inspiring package tcR). From the text it is not immediately clear why there is the need for a brand new package rather than a simpler 'wrapper' to bridge the gap between single cell based data and existing analytical packages.**

 Thank you for your input. To clarify, indeed a gap exists. The vast majority of packages available for V(D)J single-cell analysis is python-based reconstructions of TCR/BCR sequences (see, scTCRseq , TRAPeS and TraCeR). The tcR package that the reviewer indicates is in fact deprecated and only functional for bulk sequencing, there is no barcoded-based method. However, the authors of tcR have a newer R package, immunarch, that is freely available and produced by the company immunomind. This newer package offers single-cell support, but the workflow within the package does not allow for the combination of both TCR/BCR chains, and will call clonotypes with a single chain. In addition, as the reviewers keenly mention in the accompanying comments, single-cell technologies have unique pitfalls that need to be addressed as opposed to just creating wrapper functions. These concepts have been added to a newer version of the manuscript.

**Although package functionalities are clearly illustrated in the paper, software documentation could be improved: behaviour of some functions is not immediately clear without studying the code directly (e.g. the way multi-chains clonotypes are treated by combineContigs()).**

 We have added extensive documentation to the package.

**Similar to its inspiring package (tcR) and leveraging on the vegan package, scRepertoire implements different measures for diversity. Nonetheless, it has been suggested that conventional diversity measures fail to describe properly clonotype distributions and several complementary methods have been proposed like the Recon package by Kaplinsky and Arnaout** **or Startrac package by Zhang and colleagues. Interaction with such packages would greatly increase scRepertoire analytical effectiveness.**

 We have added the function StartracDiversity() to the development version of the package to allow for the migr, exp, and tans indices to be calculated from the Seurat object after the addition of the clonotype information. This function is detailed in the vignette due to limited word count of this article.

**Similarly, the function abundanceContig(), especially in the unscaled form, is of little use per se. Interestingly, recent methods have been proposed to for the comparative analysis of clone size distributions and could be easily incorporated into scRepertoire adding considerable power to it (though assessment of required numerosity should be introduced).**

 We have added the function clonesizeDistribution() to allow users to examine the hierarchical  clustering based on clone size distributions of samples to the scRepertoire package along with a comprehensive description and covered the usage in the vignette accompanying the package. Due to the length limitation for the manuscript, we only reference the function in the manuscript with the appropriate citation.

**The package claims to be designed both for TCR and BCR analysis but definition of clonality in B cells is slightly different than in T cells due to isotype switch and somatic hypermutation phenomena following activation. Therefore clonotype identity between two cells should be defined differently between BCR and TCR analysis.**

 We agree with the need to add thresholding for clonotype assignment for the BCR in the context of somatic hypermutation. We have separated the combineContig() function into combineTCR() and combineBCR() functions, the latter organizes the data similarly, but defines nucleotide sequences by the normalized Hamming Distance, with sequences greater than >0.85 assigned to the same group. In the strict definition of the clonotypes in the combineBCR() the vgene is also added to both the light and heavy chains. We have kept the ability to visualize and analyze clonotypes at the amino acid, nucleotide sequence and gene level as well, to all users more choices.

**Concerning overlap, beside the nice representation as a heatmap, it could be useful to have the chance to output the matrix itself rather than the plot only.**

 When appropriate, the visualization functions have the exportTable variable that allows a user to get the table or matrix that enables the visualization. Although this feature is already present, we will add discussion to the manuscript and accompanying vignette.

**10x vdj methods occasionally fails to reconstruct complete clonotypes or it reconstructs putatively aberrant clonotypes (clonotypes with multiple beta chains). Currently scRepertoire does not allow to filter for specific chain compositions but such feature would be worth adding, together with a graphical visualization of relative frequencies of chain composition across clonotypes.**

 If the authors understand the suggestion, the reviewers are asking for additional filtering options for combineTCR() and combineBCR(), two separate functions created from the reviewers earlier points. The function has the ability to remove barcodes with NA values or with multiple chains. More specifically to the combineTCR() function we have added removeMulti (removes all barcodes with >= 3 chains) and filterMulti (selects the 2 highest expressing chains). In terms of the chain filtering and visualizations, we are more than happy to add additional filtering and visualization tools for users. If the reviewer could clarify more on the how they envisioned the chain composition visualization, we would appreciate more insight.

**Paired 10x gene expression profile and vdj scoped analysis are not guaranteed to reconstruct the information for the exact same pool of barcodes, thus the combineSeurat() function could be improved by allowing to specify whether an inner or Seurat-sided joining is to be performed and ensuring that the joining is performed correctly.**

 We have added the variable NA into the new combineBCR() and combineTCR(), which functions by removing cells without clonotype information. If we understand the reviewer correctly, the differential joining function would also need to address the seurat object as a whole instead of just merging the meta.data.
